# Vaccine manufacturing capacity in low- and middle-income countries

**DOI:** 10.2471/BLT.20.273375

**Published:** 2021-07-01

**Authors:** Mohammed Imran Khan, Aamer Ikram, Hasan Bin Hamza

**Affiliations:** a PHC Global, 241 Bahadur Shah Zafar Road, Bahadurabad 3, Karachi Pakistan 74800, Pakistan.; b National Institute of Health, Islamabad, Pakistan.; c Health Planning, System Strengthening, Information Analysis Unit, Ministry of National Health Services, Regulations and Coordination, Islamabad, Pakistan.

Vaccination is the most cost-effective public health intervention for the control and eradication of infectious diseases, including the coronavirus disease 2019 (COVID-19).

Ensuring equitable access to all vaccines is needed, particularly now with the significant global demand for vaccine doses to contain the severe acute respiratory syndrome coronavirus 2 (SARS-CoV-2). However, the focus on ensuring the availability of COVID-19 vaccines has affected the manufacturing and supply chain of other vaccines. In addition, the COVID-19 pandemic has resulted in a drop in health service use including immunization services.^1^ This drop will likely lead to an increase in the risk of vaccine-preventable infectious disease outbreaks.^2^

The support from Gavi, the Vaccine Alliance and other global donors through COVAX has been instrumental in providing SARS-CoV-2 vaccines to low-income countries; however, global partners face limitations in meeting vaccine needs. As of 10 June 2021, 2.2 billion doses of COVID-19 vaccine have been administered, but most of these vaccines are from manufacturers based in high-income countries,^3^ and delays in vaccine availability have been more pronounced in low- and middle-income countries. Additionally, some countries are either not supported through Gavi because they do not meet the eligibility criteria or cannot allocate enough financial resources to purchase vaccines.

Timely and wider access to vaccines could be facilitated through local vaccine manufacturing. Countries such as Brazil, Cuba, India, Indonesia and Pakistan have public sector vaccine manufacturing and can therefore make independent decisions on vaccine manufacturing and supply, provision and introduction in their respective health systems. Vaccine development and manufacturing is laborious, requiring not only technological capacity but also financial support from governments.^4^ Many low-income countries have not been able to establish their own manufacturing units because of their human, financial and technical resource constraints in vaccine manufacturing. These countries allocate minimal resources to health, and in the presence of many other needs, vaccine research, development and manufacturing becomes a low priority. This situation has resulted in the slow decline of public-sector vaccine manufacturing capacity except for those countries where health is a priority, or where the governments decide to collaborate with the private sector to manufacture vaccines.

Currently, four vaccine manufacturers control about 90% of the global vaccine market,^5^ which presents a twofold challenge. First, vaccine development prioritization is driven by cost–effectiveness and impact analysis centred on the global burden of diseases, vaccine supply and population coverage. Second, with only a few groups involved in vaccine development and manufacturing, access to vaccines in low- and middle-income countries is dependent on the inclination of the leads in the industry. Except for a few diseases such as yellow fever and Japanese encephalitis, many other diseases that may have significant impact on a smaller geographical population fall low on the priority list of global vaccine manufacturers and financiers.^6^ Rabies vaccine, and until recently, typhoid vaccine, are good examples of the influence that regional high-burden diseases can have on global vaccine manufacturing policy.^7^

National public sector or public–private partnership for vaccine manufacturing have many benefits. The countries that establish national vaccine manufacturing will have the autonomy to decide which vaccines and what combination of antigens they produce. The cost of the vaccine will be much lower than the price governments or global partners currently pay to the vaccine manufacturers. While setting up vaccine manufacturing units and supply may be costly, a national facility would have the advantage of focusing on the vaccines needed based on local epidemiology and impact.^8^ Often, vaccines target pathogen strains based on the country where vaccines were developed and thus may not be as effective in other countries or areas.^9^ These issues can be addressed through in-country manufacturing. Countries can save the cost of vaccine procurement and reach populations more quickly. A national facility would also create an environment for vaccine and infectious disease research.

While the global community has set up a platform, the Access to COVID-19 Tools (ACT) Accelerator, which includes COVAX, populations in the vaccine-manufacturing countries will benefit early while others will have to wait. Among other lessons, one to take away from the pandemic is that low-income countries should build the capacity for in-country vaccine manufacturing to address local vaccine requirements and be better prepared for the next health challenge.
